# A machine learning strategy to identify candidate binding sites in human protein-coding sequence

**DOI:** 10.1186/1471-2105-7-419

**Published:** 2006-09-26

**Authors:** Thomas Down, Bernard Leong, Tim JP Hubbard

**Affiliations:** 1 Wellcome Trust Sanger Institute, Hinxton, Cambridge CB10 1SA, UK

## Abstract

**Background:**

The splicing of RNA transcripts is thought to be partly promoted and regulated by sequences embedded within exons. Known sequences include binding sites for SR proteins, which are thought to mediate interactions between splicing factors bound to the 5' and 3' splice sites. It would be useful to identify further candidate sequences, however identifying them computationally is hard since exon sequences are also constrained by their functional role in coding for proteins.

**Results:**

This strategy identified a collection of motifs including several previously reported splice enhancer elements. Although only trained on coding exons, the model discriminates both coding and non-coding exons from intragenic sequence.

**Conclusion:**

We have trained a computational model able to detect signals in coding exons which seem to be orthogonal to the sequences' primary function of coding for proteins. We believe that many of the motifs detected here represent binding sites for both previously unrecognized proteins which influence RNA splicing as well as other regulatory elements.

## Background

In eukaryotes, the majority of transcripts are processed by splicing to remove intron sequences and form a mature messenger RNA. There are well established conserved sequence motifs at the intron/exon and exon/intron boundaries that are known to be recognised by the splicing machinery. However, even for short intron sequences, it has been concluded that these signals do not contain sufficient information to accurately define splice sites [1] and that other splicing factors, with associated binding sites, must be involved.

There is evidence that splicing is partly promoted and regulated by sequences embedded within exons. A number of sequences have been found embedded in the exons of both viral and cellular genes which can promote or repress the utilization of alternative splice sites. These are usually purine-rich sequence located near an alternative splice donor, that bind splicing factors such as members of the SR family. SR proteins are highly conserved serine/arginine-rich RNA-binding proteins (For review, see [2]). They are essential splicing factors and have been shown to regulate the selection and use of alternative splice sites [3-10]. It is known that they function very early in the spliceosome assembly process, promoting the binding of U1 snRNP to the splice donor and of U2AF to the splice acceptor, apparently by interacting with U1 70 K and U2AF respectively. Observations have shown that SR proteins bound to exons recruit splicing factors to the adjacent splice sites. Nine human SR proteins are currently known: SF2/ASF [2,4,6,7], SC35 [4,6-8,11,12], SRp20 [6,8], SRp40 [4], SRp55 [4], SRp75 [2], SRp30c [2], 9G8 [6,9,11] and the divergent SRp54 [2]. These proteins are closely related in sequence and structure and share the ability to activate splicing. Human SR related proteins of the Tra2 family are similarly known to be splicing regulators and sequence specific activators of pre-mRNA splicing [8].

Early research concentrated on how SR proteins function to regulate alternative splicing. However, the binding of SR proteins to constitutive exons, which are included in all splice variants of a gene, is also thought to play an important role in splicing. The exon definition model proposes that interactions between components bound to splice sites flanking an exon serve to highlight usually small exons against a background of much larger introns. It is conjectured that the majority of constitutively spliced exons are defined by this mechanism. To support the model, a number of SR protein binding sites have been identified in constitutive exons, and shown to function as constitutive splicing enhancers [6,13].

Although these binding sites are believed to be common, studying their sequences is difficult because they are embedded in exons, most of which are also functional protein-coding sequences. When a particular motif is found to be over- or under-represented in coding exons, it is generally unclear whether it is a consequence of the underlying protein sequence, or an unrelated signal, such as a splice enhancer, embedded in the protein coding sequence. Here we describe a novel strategy for resolving this uncertainty. Starting with annotated coding exons, we generate a 'neutralized' exon set: sequences which are generated randomly, but which nevertheless preserve both the amino acid sequence and overall composition features of the original exons. We then apply machine learning software to compare the original and neutralized exons. Since the neutralized set codes for the same proteins, it is likely that any feature which can be used to discriminate between the original and neutralized sets is performing some function which is independent of the exons' primary, protein-coding, function.

## Results and discussion

### Neutralized exons

**Figure 1:**
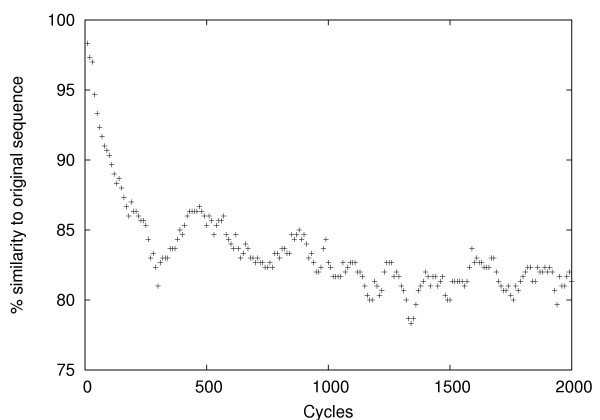
**A plot of the time-course vs percentage of sequence identity for neutralizing a typical 300 base sequence**. A plot of the time-course vs percentage of sequence identity for neutralizing a typical 300 base sequence. The level of sequence identity falls in a steady rate for about 500 cycles and then comes to a minimum at about 1000 cycles. From 1000 cycles onwards, there are only slight fluctuations which implies that 1000 cycles of neutralization is the optimum value to randomize any sequence with a length up to 300 bases.

9091 internal coding exons with lengths ranging from 100 to 300 bases were extracted from the Vega database of annotated human genomic sequence [14]. Testing the neutralization process (see methods section) on a typical 300 base exon (figure 1) we see that the sequence identity between the original and neutralised sequence falls steadily for approximately 500 cycles, then stabilizes and only fluctuates slightly for the remainder of the cycles. Allowing some margin for exceptional sequences, this suggests that that 1000 cycles of neutralization is adequate to randomize any sequence with a length up to 300 bases. Running the neutralization algorithm on the complete set of exons, for 1000 cycles per exon, gave a neutralized set with an average of 78% sequence identity compared to the reference set. The average dinucleotide compositions of the exons before and after neutralization is shown in table 1. We can see that most dinucleotides show negligible change in composition during the neutralization procedure, and in the most extreme case (the tt dinucleotide), the proportion of the sequences composed of tt dinucleotides changes by less that 2%. Therefore, the neutralization algorithm seems able to preserve overall sequence composition while substantially changing the sequence itself.

**Table 1:** Comparison of dinulceotide frequencies in original and neutralized exons

Dinucleotide	Original exons	Neutralized exons
aa	7.71%	7.73%
ac	5.56%	5.55%
ag	8.19%	8.21%
at	5.58%	5.62%
ca	8.06%	8.06%
cc	7.17%	7.18%
cg	2.73%	2.74%
ct	6.96%	6.88%
ga	7.76%	7.76%
gc	6.41%	6.38%
gg	6.61%	6.54%
gt	4.55%	4.58%
ta	3.43%	3.47%
tc	5.72%	5.69%
tg	7.98%	7.96%
tt	5.48%	5.58%

Comparison of dinucleotide frequencies in original and neutralized exons. In all cases frequencies before and after neutralization are very similar.

### Motif-based models can effectively distinguish between original and neutralized exons

From both the original and neutralized sets, we removed 300 randomly selected sequences for use as test data. The remainder were used to train a Convolved Eponine Windowed Sequence (C-EWS) model (see methods section and [15]). These models are based on scaffolds of one of more sequence motifs (in this case, limited to a maximum of three per scaffold). The motifs are represented as DNA weight matrices [16]. When a scaffold includes more than one motif, probability distributions associated with each motif indicate the preferred relative positions of those motifs. Each scaffold has an associated weight, which is used to combine scaffold scores in a relevance vector machine.

**Figure 2:**
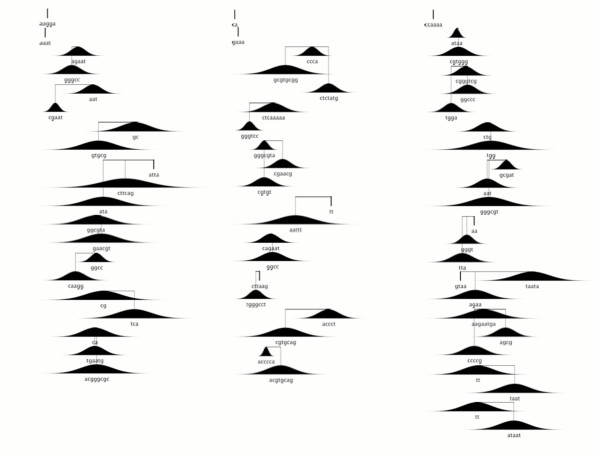
**Positively weighted scaffolds in the Eponine-Exons model**. Positively weighted scaffolds in the Eponine-Exons model. Motifs that together form a scaffold are linked by lines. Scaffolds are generally composed of 1, 2 or 3 motifs. The black filled areas show the relative position distributions (histograms) of the motifs within a scaffold, while the sequences represent the most likely base at each position in the weight matrix of the motif.

**Figure 3:**
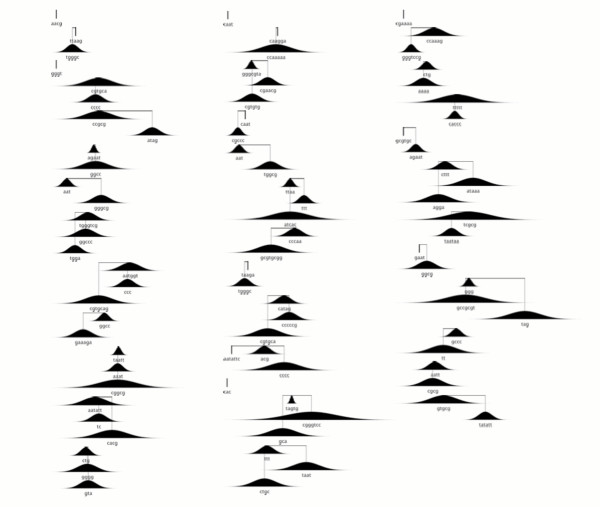
**Negatively weighted scaffolds in the Eponine-Exons model**. Negatively weighted scaffolds in the Eponine-Exons model. The interpretation of the whole figure is similar to figure.

Training resulted in a complex model consisting of 216 scaffolds, split evenly between positively-weighted scaffolds – signals which are likely to be over-represented in the original exons – and negatively weighted scaffolds. The complete set of scaffolds can be seen in figures 2 and 3.

**Figure 4:**
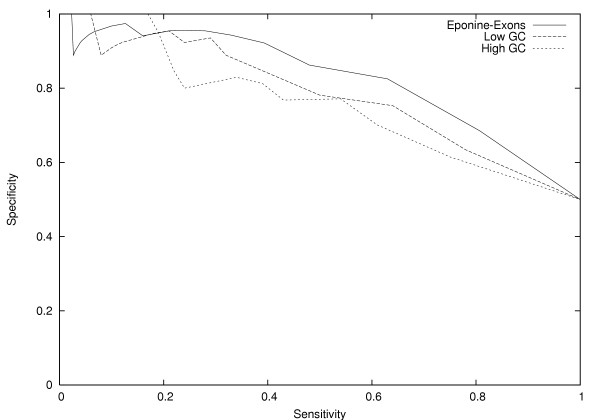
**Specificity vs sensitivity plot for the Eponine-Exons model**. A plot of specificity *vs*. sensitivity showing the ability of the eponine model to discriminate between unseen original and neutralized exons. Plots are shown for the whole test set and two sets split between low and high GC content.

We tested the resulting model's classification ability using the unseen test data. Specificity (proportion of predictions that are correct) and sensitivity (proportion of original exons detected) are shown for a range of classifier score thresholds in figure 4. From the sensitivity-specificity curve, we see that the Eponine-Exons model is effective in distinguishing between original and neutralized exons.

Since the human genome is a mosaic of isochores of high and low GC content and neutralization generates sequences with average properties, it was possible that the classifier's performance was an artifact resulting from differences in GC distribution between original and neutralized sequences. To check this was not the case, the original set of sequences was split into subsets of high and low GC content. For each subset an associated neutralized set was generated, based on the average properties of that subset. Separate models were then trained and tested (see figure 4). We find that for both high and low GC subsets the classifier is still able to distinguish original and neutralized sequences.

### The Eponine Exons model can also distinguish non-coding exons from randomized sequences

Since the negative training set is not composed of natural sequences, an obvious concern is that the features we have detected are artifacts of the neutralization procedure, and are of no use when analysing real sequence data. To validate the Eponine-Exons model, we tested it on additional sequences from four classes: protein coding exons not in the original training set, non-coding (UTR) exons, introns, and intergenic regions, all from annotation of finished human chromosomes obtained from chromosomes 9 and 10 in the Vega database [14]. For exons, introns and intergenic regions, we extracted sets of 1000 sequences, each of 200 bases long (one set each for exons and introns, four independent sets for intergenic regions). We were unable to obtain sufficient 200 base non-coding exonic sequences, so instead we used 100 base sequences.

**Figure 5:**
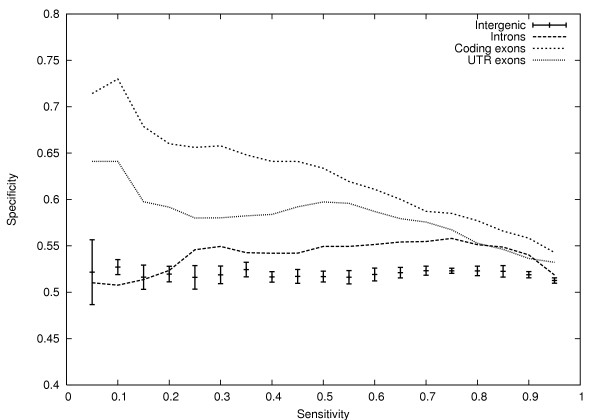
**Sensitivity-specificity curves for Eponine-Exons model on independent sets of sequences**. Specificity *vs*. sensitivity for the Eponine-Exons model on independent sets of intergenic, intron, and UTR exon sequences compared with random sequences of matching mono- and di-nucleotide composition. All curves are based on sets of 1000 sequences. In the case of intergenic sequences, standard-deviation error bars were calculated based on results from four independent sets of sequences.

For each data set, we produced a corresponding set of negative sequences with matching mono- and di-nucleotide composition using the randomizing procedure detailed in the methods section. We then used the Eponine-Exons model as a classifier, and tested its ability to separate each of the positive sequence sets from its corresponding negative sequence set. Sensitivity-specificity curves are shown in figure 5.

In the case of the intergenic sequences, there is no significant discrimination between real and shuffled sequences. The coding sequences, however, could be discriminated, as might be expected from a classifier trained on protein-coding sequences. Moreover, the model was also able to distinguish many non-coding exons from their shuffled counterparts. This result is highly significant because it indicates that at least some of the signals discovered in coding exons are general to both coding and non-coding exons. This is consistent with the idea that they are involved in exon definition and splicing. Finally, there is a far weaker, but still possibility significant, discrimination between real and shuffled intron sequences. One explanation for this is that the introns were contaminated with a small number of exons which were missed during the annotation process. However, a second possibility is that, in addition to an exon-specific signal, the Eponine Exons model is also detecting some (weak) signal – perhaps an anti-termination signal – which is found throughout transcribed regions of the genome. We note that the discrimination between exons and shuffled sequences is not as effective as discrimination between exons and neutralized sequences, which implies that some degree of overfitting may have occurred in the training process. Nevertheless these results suggest that a significant amount of relevant information has been captured.

**Figure 6:**
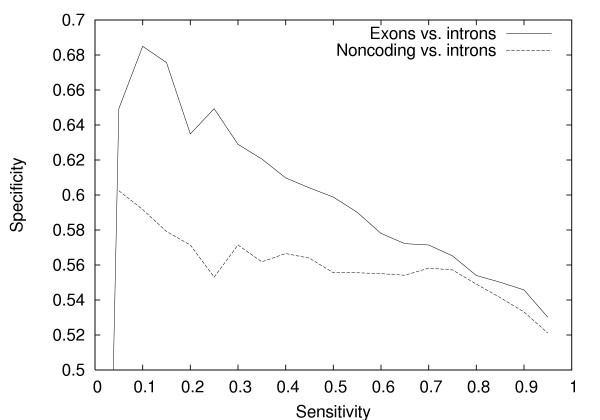
**Sensitivity-specificity curves for Eponine-Exons model to distinguish non-coding exonic sequences and intronic sequences**. Specificity *vs*. sensitivity for the Eponine-Exons model to distinguish exonic sequences (both coding and non-coding) and intronic sequences with matching GC content.

Based on these results, we hoped that the model would be able to successfully discriminate between exons and other biologically authentic sequences. However, in initial tests exons did not generally score higher than intronic or intergenic sequences (data not shown). When we inspected the sequences, we found that the exons had a significantly different composition from other sequences: in particular, they tended to have a higher GC content. We suspected that the classifier output was somewhat sensitive to the GC content of the input sequence. When we compensated for this effect by randomly sampling a subset of intronic sequences with GC contents matching that found in the exonic test sequences (both coding and non-coding), the eponine-exons model was able to discriminate between the sets (see figure 6).

Comparing introns with similar GC content to the exons, the discrimination between non-coding exons and introns is relatively poor – although still substantially better than random. This points to a limitation in the training approach of exons vs neutralized exons: splicing signals in a background of non-coding exonic sequence are not captured well by our classifier. In addition, [17] conclude the most exons require sequences in the flanks for efficient splicing, and these will clearly not be learned by our method, which focusses on the body of the exon.

The neutralization process we use has some similarities to the dicodon shuffling algorithm proposed by [18], which swaps pairs of synonymous codons under a constraint that the dinucleotide composition of the sequence must be preserved. We implemented this algorithm to compare its performance and properties with our own neutralization strategy. On the same sequences, the dicodon shuffling algorithm gives a set of sequences with an average sequence identity of 90% to the reference set. This is much higher than the 78% sequence identity observed after applying our neutralization procedure. This is unsurprising because the constraints under dicodon shuffling are stronger, requiring the dinucleotide composition to be maintained on a per-sequence basis, whereas neutralization only requires this across the complete set of sequences. To test whether this makes a significant difference when searching for functional motifs, we carried out identical training using C-EWS and obtained a model of similar complexity. However we found it had a much lower classification ability using unseen test data (*c*.*f*. figure 4) and was unable to distinguish between shuffled and reference sequences better than random for either coding, non-coding or intronic sequence (*c*.*f*. figure 5, data not shown). We conclude that our approach of neutralization destroys information more readily than dicodon shuffling and that this is necessary to reveal a strong enough signal for motifs to be recognised.

### Comparison of learned motifs with known splice-enhancer sites

If the motifs learned in the Eponine Exons model are meaningful, a subset of them might be expected to match the set of motifs that have already been show to influence splicing. We therefore compared the weight matrices in the positively weighted scaffolds of our exon model with known splice enhancer sites [2-12].

Direct comparisons of weight matrices with sequence motifs – with or without ambiguity symbols – is complicated, since different positions in a weight matrix may convey different amounts of information. Furthermore, it is not certain that either the learned weight matrices or the published motifs correspond to the full length of the biologically functional sequence. It is therefore important to consider a range of possible alignments of motifs to weight matrices.

We extracted all weight matrices from the Eponine Exons model which were associated with a positive scaffold and which were at least four bases long. For each known motif, we calculated the log-odds score against all weight matrices from scaffolds with weights greater than 1.0, considering all possible alignments with up to one base of overlap, and took the maximum score. We summed the maxima to give an aggregate score for the degree of match between that motif and the full Eponine model.

We then constructed 1000 random weight matrix sets, each with matched information profiles to the Eponine Exons matrix set. Again, we calculated the aggregate score for each known motif. Finally, we counted the fraction of random matrix sets which scored higher than the Eponine Exons matrices. This can be considered as an empirical p-value, with low values providing strong support for the hypothesis that there is some correspondance between motifs in the Eponine Exons model and the known motif being tested. Presumably, in each case there is some subset of Eponine Exons motifs which actually correspond with the known motifs, but identifying these in a statistically rigorous manner is harder: since the Eponine Exons model is relatively large, a corresponding multiple testing correction would be needed. We therefore limit ourselves to considering aggregate scores here, even though we believe it to be a rather conservative strategy.

Tables 2 and 3 list the p-values for known motifs from exons and introns respectively. We see significant associations for 4/12 exonic motifs and 2/9 intronic motifs. Interestingly, one of the two matched intronic motifs is a substring of the other, and they are believed to be binding sites for the same protein – Tra2beta – so arguably they should be merged, in which case 1/8 intronic motifs are matched. Such a preference for exonic over intronic motifs may not be surprising, since the model was training only on exonic sequences. Several other computational methods for finding splice enhancer signals have been proposed [19-22]. One method, RESCUE-ESE [19] compares the sequences around weak consensus splice sites with those around strong consensus sites, with the expectation that splice enhancer motifs are more likely to be found in the vicinity of weak splice sites. This strategy relies information orthogonal to that used by our method, thus it is interesting to compare the results. We compared the RESCUE-ESE derived motifs with the eponine model using the same approach as used for the experimentally determined motifs (see above). The results in table 4 shown that our model finds one of the motifs detected by the RESCUE-ESE method. We are not surprised that our method did not find all the RESCUE-ESE motifs since RESCUE-ESE is designed to detect both exon and intron localized motifs.

**Table 2:** Comparison of known ESE motifs which are located inside the internal exons.

Consensus	SR Protein or Gene	p-value	Status	Reference
aggacagagc	ASF/SF2	0.398		Tacke et al (1995)
aggacgaagc	ASF/SF2	0.007	+	Tacke et al (1995)
rgaagaac	ASF/SF2	0.010	+	Tacke et al (1995)
acgcgca	ASF/SF2	0.062		Tacke et al (1995)
aggacrragc	ASF/SF2	0.117		Tacke et al (1995)
tscgkm	SRp55	0.096		Liu et al (1998)
cctcgtcc	SRp20	0.948		Tacke et al (1999)
tgttcsagwt	SC35	0.707		Tacke et al (1999)
tgcngyy	SC35	0.990		Schaal et al (1999)
acgaggay	9G8	0.005	+	Cavaloc et al (1999)
tcwwc	dsx	1.000		Schaal et al (1999)
aggagat	SC35	0.020	+	Cavaloc et al (1999)

Comparison between the Eponine Exons model and known ESE motifs which are located within internal exons. A '+' in the status column indicates a significant p-value <= 0.05.

**Table 3:** Comparison of known ESE motifs which are located at the splice acceptor site of the exon-intron boundaries

Consensus	SR Protein or Gene	p-value	Status	Reference
ctcktcy	SRp20	0.922		Schaal et al (1999)
rgaccgg	SC35	0.054		Schaal et al (1999)
agagcagg	ASF/SF2	0.135		Zheng et al (1999)
rgackacgay	9G8	0.352		Tian et al (1999)
aagaagaa	Tra2 (beta)	0.015	+	Tacke et al (1995)
tcaaca	Tra2	0.904		Lynch et al (1996)
gaagaa	Tra2 (beta)	0.010	+	Tacke et al (1999)
gacgacgag	Pu1	0.111		Bourgeois (1999)
gatgaagag	Pu2	0.183		Bourgeois (1999)

Comparison between the eponine motif model and known ESE motifs which are located in introns at the splice acceptor site of the exon-intron boundaries. A '+' in the status column indicates a significant p-value <= 0.05.

**Table 4:** Comparison of known ESE motifs with RESCUE-ESE method

Consensus	p-value	Status
atcttc	0.993	
actaca	0.997	
ttggat	0.415	
gaatca	0.213	
gaagaa	0.008	+
ttcaga	0.898	
gacaaa	0.239	
ctgaag	0.548	
aatcca	0.952	
aacttc	0.813	

Comparison between the eponine motif model and known ESE motifs determined using the RESCUE-ESE method in [19]. A '+' in the status column indicates a significant p-value <= 0.05.

Finally, we performed an analogous experiment to discover what proportion of our discovered motifs matched previously known motifs. In this case, 41% of motifs from the Eponine-Exons model had a good match to at least one motif from the exonic splice enhancer and RESCUE-ESE sets described above (log-odds score at least 0.2 above the expected score based on a set of 1000 shuffled motifs), suggesting that around 59% of discovered motifs may be novel.

## Conclusion

We have shown that a motif-based machine learning strategy can extract signals which discriminate effectively between original and neutralized sets of protein-coding exons. The resulting model included recognizable consensus sequences for many of the previously reported splice-enhancer binding sites.

Although the model was trained only on coding exon sequences, it gives high scores for both coding and non-coding exons, but not introns or intergenic regions. We therefore believe that the neutralization strategy is a powerful and effective method for learning functional non-coding elements embedded in protein coding sequence.

One interesting feature of the model learned here is its complexity: 216 scaffolds, split evenly between positively and negatively-weighted scaffolds. This is a large number, both in absolute terms, and also in comparison with EWS and C-EWS models trained for other purposes, such as promoter prediction [23]. This suggests that a large number of functional elements could play widespread roles in exon definition. The motifs learned here which cannot be assigned to any currently known splice-regulating protein are strong candidates for investigation with a view to discovering novel splice regulators.

We hope that changes in the machine learning strategy will improve the classification accuracy of this method. Possible candidates for investigation include the use of scaffolds comprising more than 3 motifs, and the replacement of simple weight matrices with more complex models which serve as better representations of protein binding sites. We do not, however, necessarily expect that it will be possible to classify original and neutralized exons with 100% accuracy: most proteins can accept many mutations with little or no change to structure and function, so it is inevitable that some of the information which the cell uses to define exons will be encoded in the choice of amino acids, rather that just the choice of nucleotides used in redundant positions.

In the future, we hope to apply the results of this technique to the problem of *ab initio *prediction of genes. Current gene-prediction techniques rely on a combination of splice-site models and 'coding bias' – the observation that coding sequence looks very different from intronic and intergenic sequence when considering properties such as hexamer frequencies. While such methods work reasonably well for protein-coding genes, they seldom make good predictions of untranslated regions, and do not detect the non-coding RNA genes which are now known to be important in many aspects of cellular function. Scanning bulk genomic DNA using our model makes many predictions outside known exons (*i.e*. a high apparent false positive rate). This suggests that while the motifs discovered here may be necessary for efficient splicing, they are not sufficient to fully define exons. We hope that building knowledge of candidate binding sites into gene prediction methods, together with other features such as splice junction consensus sequence, will improve the prediction of all spliced transcripts, whether coding or non-coding.

## Methods

### Genome sequence and annotation

Curated annotation of gene structures on chromosomes 6, 13, 14, 20, and 22 were obtained from the Vertebrate Genome Annotation (Vega) database [14]. We extracted a total of 27954 internal protein-coding exons of different intron phases for our positive training set. Based on the definition [24], an intron contained within CDS is said to have a phase of zero if the intron demarcates a codon boundary, a phase of one if it divides the codon between the first and second nucleotides, and a phase of two if the intron divides a codon between the second and third nucleotides. The position of an exon with respect to the codon positions can be defined by the phases of upstream and downstream flanking introns and when an exon is flanked by introns of the same phase, it will be a multiple of three nucleotides in length. The phase definition is important for the neutralization scheme described in section.

Vega data, which is stored in an Ensembl style database [25] was extracted directly from the database using the BioJava toolkit with biojava-ensembl extensions [26].

### Constructing a non-redundant set of sequences

To eliminate similar sequences from the datasets, we performed an all-against-all comparison of the sequences using NCBI blastn [27] using default options (word size 11, match reward +1, mismatch penalty-3) and recorded all pairs with a bit score ≥ 35. We then performed single-linkage clustering, and from each cluster we picked one member at random to represent that cluster in the final data set.

### Neutralization of coding sequences

Exon neutralization is a process which randomizes the sequence of a set of protein-coding exons while maintaining three key constraints:

• The neutralized exons code for the same protein sequence as the real exon

• The frequency of a particular codon being used to represent a particular amino acid is maintained

• The overall dinucleotide composition of the set is maintained

Thus, by comparing neutralized exons against the corresponding set of original exons, it should be possible to detect sequence features which are preferentially over- or under-represented in the original exon set due to the amino acid codons used. Features that occur purely as artifacts of the underlying protein sequence will occur with equal frequency in the original and neutralized sets. Because the differences in codon usage are subtle, a large data set is required to construct a representative model. However, the curated human gene set is sufficiently large for this purpose.

The neutralization process used here is a Monte-Carlo method, whereby small (single-codon) changes to the sequence are proposed, then accepted or rejected on the basis of a probabilistic model which captures the features listed above. In this case, the model is encapsulated as a set of *conditional codon usage tables*. Consider a codon *C *which encodes amino acid *A*, and is flanked by nucleotides *p *and *q *to form the pentanucleotide *pCq*. Our model records the probability of the codon being used in this context:

*P*(*C*|*A*, *p*, *q*)     (1)

The model is initialized for a given set of exons by simply counting all in-frame codons in the exon set. For each exon in the set, a number of neutralization cycles are performed. In each cycle, one in-frame codon position within the exon is chosen at random. Let *C *equal the current codon at this position. If it encodes an amino acid which has only a single codon in the universal genetic code, it is always left unchanged. Otherwise, a synonymous codon, *C'*, is proposed by sampling from a uniform random distribution over all synonyms, *Q*(*C'*|*C*). Next, the appropriate conditional codon usage table is consulted, given the two bases either side of *C*. We accept or reject the proposed change on the basis of the Metropolis-Hastings criterion [28]:

z=P(C′)P(C)Q(C′|C)Q(C|C′)     (2)
 MathType@MTEF@5@5@+=feaafiart1ev1aaatCvAUfKttLearuWrP9MDH5MBPbIqV92AaeXatLxBI9gBaebbnrfifHhDYfgasaacH8akY=wiFfYdH8Gipec8Eeeu0xXdbba9frFj0=OqFfea0dXdd9vqai=hGuQ8kuc9pgc9s8qqaq=dirpe0xb9q8qiLsFr0=vr0=vr0dc8meaabaqaciaacaGaaeqabaqabeGadaaakeaacqWG6bGEcqGH9aqpdaWcaaqaaiabdcfaqjabcIcaOiqbdoeadzaafaGaeiykaKcabaGaemiuaaLaeiikaGIaem4qamKaeiykaKcaamaalaaabaGaemyuaeLaeiikaGIafm4qamKbauaacqGG8baFcqWGdbWqcqGGPaqkaeaacqWGrbqucqGGOaakcqWGdbWqcqGG8baFcuWGdbWqgaqbaiabcMcaPaaacaWLjaGaaCzcamaabmGabaGaeGOmaidacaGLOaGaayzkaaaaaa@47FE@

When *z *≥ 1, the codon substitution is always accepted, when *z *< 1 the substitution is accepted with probability *z*. In this case, at any given position, the proposal distribution *Q *is always uniform, the second term of this expression can be ignored: it is simply the fit of the proposed new codon to the model represented by the conditional codon usage tables which is important.

### Generating random sequences with matching mono- and di-nucleotide composition

In order to randomize a set of sequences while maintaining mono- and di-nucleotide compostion, the sequence set is first analysed and its initial dinucleotide composition recorded. Then a large number (typically 500) of iterations are performed. For each iteration two points within the sequence are selected at random, breaking it into three segments, *ABC*. A rearrangement to give the sequence *BAC *is then proposed. This rearrangement destroys two dinucleotide pairings and creates two new pairings. The probabilities of the sequences *ABC *and *BAC *are calculated from the dinucleotide frequency table, and the rearrangement is accepted or rejected based on the Metropolis-Hastings criterion described above.

### Generating sequences with matching GC content

For a further test in using the eponine-exons model in distinguishing non-coding exons and introns, we took our set of non-coding exons (trimmed to 100 bases long) and calculated the histogram of GC content. Then we shredded a large set of introns into 100 base fragments and randomly sampled fragments with a matched histogram of GC content like the exons. The Eponine-Exons model can successfully discriminate between exons and this GC matched set of intronic sequences. We have also done the same for the set of coding exons and a corresponding set of intronic sequences with similar GC content distribution.

### The Convolved Eponine Windowed Sequence model

Sequence classification models were trained using a Relevence Vector Machine (RVM) [29]. The RVM is a method for learning sparse classification or regression models by optimizing the weights applied to an arbitrary set of basis functions. The final model output for some piece of data is the weighted sum of basis function outputs. The RVM's sparsity property means that it will generally select only a subset (often a small subset) of the basis functions provided for the final model, with all others given zero weights and thus making no contribution. Sparsity is generally considered a desirable feature in a machine learning system [30]. This is consistent with the intuition that a simple model is more likely to make useful generalizations which can be applied to unseen data, rather than solving the problem 'trivially' by memorizing the training data. Sparse training methods also offer a partial solution to the question of feature selection. The RVM is named by analogy to the better-known Support Vector Machine method, which is also a kind of sparse trainer, and indeed the RVM was initially presented as an alternative and direct competitor to the SVM. However, while the SVM can only build models that can be expressed using a suitable kernel function, the RVM can build any model that can be expressed as a sum of basis function outputs: we therefore consider it a more flexible method. In this case, we use the RVM as an engine to drive the construction of a motif-oriented sequence model.

The Convolved Eponine Windowed Sequence (C-EWS) model is a classification model for small regions (windows) of sequence data. In this case, each basis function defines a scaffold consisting of one or more DNA Position-Weight Matrices (PWMs), each with an associated position distribution relative to a scaffold anchor point. In principle obvious choices for smooth position distributions, such as the discretized Gaussian, extend to infinity, but in practice it is reasonable to apply some cut-off: for instance, only considering the portion of the distribution which includes 99% of the total probability mass. The probabilities of all points outside this region are assumed to be infinitesimal and ignored. Now that the distributions have finite size, for a given scaffold there is a pair of integers, *n *and *m*, such that when the scaffold anchor is placed in the interval [*n *: *m*], the non-infinitesimal parts of all the position distributions fall entirely within the length of a particular target sequence. In this model, we take motifs into account regardless of where they fall on the sequence, so we sum scores from along the length of the sequence. The basis functions for the RVM therefore take the form:

φ(S)=Z∑i=nm(∏k=1K(∑j=−∞∞Pk(j)Wk(Si+ji+j+|Wk|)))     (3)
 MathType@MTEF@5@5@+=feaafiart1ev1aaatCvAUfKttLearuWrP9MDH5MBPbIqV92AaeXatLxBI9gBaebbnrfifHhDYfgasaacH8akY=wiFfYdH8Gipec8Eeeu0xXdbba9frFj0=OqFfea0dXdd9vqai=hGuQ8kuc9pgc9s8qqaq=dirpe0xb9q8qiLsFr0=vr0=vr0dc8meaabaqaciaacaGaaeqabaqabeGadaaakeaaiiGacqWFgpGzcqGGOaakcqWGtbWucqGGPaqkcqGH9aqpcqWGAbGwdaaeWbqaamaabmGabaWaaebCaeaacqGGOaakdaaeWbqaaiabdcfaqnaaBaaaleaacqWGRbWAaeqaaOGaeiikaGIaemOAaOMaeiykaKIaem4vaC1aaSbaaSqaaiabdUgaRbqabaGccqGGOaakcqWGtbWudaqhaaWcbaGaemyAaKMaey4kaSIaemOAaOgabaGaemyAaKMaey4kaSIaemOAaOMaey4kaSIaeiiFaWNaem4vaC1aaSbaaWqaaiabdUgaRbqabaWccqGG8baFaaGccqGGPaqkcqGGPaqkaSqaaiabdQgaQjabg2da9iabgkHiTiabg6HiLcqaaiabg6HiLcqdcqGHris5aaWcbaGaem4AaSMaeyypa0JaeGymaedabaGaem4saSeaniabg+GivdaakiaawIcacaGLPaaaaSqaaiabdMgaPjabg2da9iabd6gaUbqaaiabd2gaTbqdcqGHris5aOGaaCzcaiaaxMaadaqadiqaaiabiodaZaGaayjkaiaawMcaaaaa@6ABD@

where Sij
 MathType@MTEF@5@5@+=feaafiart1ev1aaatCvAUfKttLearuWrP9MDH5MBPbIqV92AaeXatLxBI9gBaebbnrfifHhDYfgasaacH8akY=wiFfYdH8Gipec8Eeeu0xXdbba9frFj0=OqFfea0dXdd9vqai=hGuQ8kuc9pgc9s8qqaq=dirpe0xb9q8qiLsFr0=vr0=vr0dc8meaabaqaciaacaGaaeqabaqabeGadaaakeaacqWGtbWudaqhaaWcbaGaemyAaKgabaGaemOAaOgaaaaa@30C0@ denotes a subsequence from *i *to *j*, *P*_*k *_is the *k*'th position distribution and *W*_*k *_is the *k*'th weight matrix in the scaffold. *Z *is the normalizing constant:

Z=4∑k=1K|Wk|m−n+1     (4)
 MathType@MTEF@5@5@+=feaafiart1ev1aaatCvAUfKttLearuWrP9MDH5MBPbIqV92AaeXatLxBI9gBaebbnrfifHhDYfgasaacH8akY=wiFfYdH8Gipec8Eeeu0xXdbba9frFj0=OqFfea0dXdd9vqai=hGuQ8kuc9pgc9s8qqaq=dirpe0xb9q8qiLsFr0=vr0=vr0dc8meaabaqaciaacaGaaeqabaqabeGadaaakeaacqWGAbGwcqGH9aqpdaWcaaqaaiabisda0maaCaaaleqabaWaaabmaeaacqGG8baFcqWGxbWvdaWgaaadbaGaem4AaSgabeaaliabcYha8badbaGaem4AaSMaeyypa0JaeGymaedabaGaem4saSeaoiabggHiLdaaaaGcbaGaemyBa0MaeyOeI0IaemOBa4Maey4kaSIaeGymaedaaiaaxMaacaWLjaWaaeWaceaacqaI0aanaiaawIcacaGLPaaaaaa@45BE@

with |*W*| denoting the length of weight matrix *W*. For the special case of scaffolds only containing a single motif, this formulation is equivalent to the Eponine Windowed Sequence model described in [15]. However, placing the motifs in scaffolds opens the possibility of learning some longer-range structural information. Groups of motifs that are regularly found together might suggest RNA-binding factors – each recognising its own short target motif – binding cooperatively.

To train C-EWS models, the RVM is first initialized with a set of single-motif scaffolds, each having a preference for some randomly picked 5 base motif. The RVM training process is then started and scaffolds which are not helpful for the classification problem are discarded. Periodically during training, the RVM working set is topped up with additional scaffolds obtained by applying one of the following sampling strategies to a scaffold (or, in some case, pair of scaffolds) randomly selected from the current working set:

• Generate a new weight matrix in which each column is a sample from a Dirichlet distribution with its mode equal to the weights in the corresponding column of the parent weight matrix.

• Generate a new weight matrix one column shorter than the parent by removing either the first of the last column.

• Generate a new weight matrix with an extra column at either the start or the end, biased in favour of a random base.

• Combine the sets of motifs from two scaffolds, with randomly chosen offsets between the two (up to some maximum number of weight matrices per scaffold, in this case 3).

• Take a scaffold with two or more PWMs and return the scaffold with one of those PWMs (picked at random) removed

• Alter the position or width of one of the relative position distributions in a scaffold.

When an operation that acts on one motif is applied to a multi-motif scaffold, one target motif from the parent scaffold is picked at random. By applying these operations repeatedly, the method is able to explore the space of scaffolds.

## Authors' contributions

TD, BL and TH conceived and designed this study, and analysed results. TD implemented the Eponine machine learning system and BL extended the system to study the exons. BL drafted the manuscript. All authors read and approved the final manuscript.
